# Application of uniform design to evaluate the different conditions on the growth of algae *Prymnesium parvum*

**DOI:** 10.1038/s41598-021-92214-y

**Published:** 2021-06-16

**Authors:** Juan Yin, Xuyang Sun, Ruizhi Zhao, Xiaocong Qiu, Rasu Eeswaran

**Affiliations:** 1 School of Civil and Hydraulic Engineering, Ningxia University, Yinchuan, 750021 Ningxia China; 2 School of Life Science, Ningxia University, Yinchuan, 750021 Ningxia China; 3 Department of Plant Soil and Microbial Sciences, Michigan State University, East Lansing, MI 48824 USA; 4 Department of Crop Science, Faculty of Agriculture, Eastern University, Chenkalady, 30350 Sri Lanka

**Keywords:** Environmental impact, Freshwater ecology, Restoration ecology, Wetlands ecology

## Abstract

*Prymnesium parvum* is an environmentally harmful algae and well known for its toxic effects to the fish culture. However, there is a dearth of studies on the growth behavior of *P. parvum* and information on how the availability of nutrients and environmental factors affect their growth rate. To address this knowledge gap, we used a uniform design approach to quantify the effects of major nutrients (N, P, Si and Fe) and environmental factors (water temperature, pH and salinity) on the biomass density of *P. parvum*. We also generated the growth model for *P. parvum* as affected by each of these nutrients and environmental factors to estimate optimum conditions of growth. Results showed that *P. parvum* can reach its maximum growth rate of 0.789, when the water temperature, pH and salinity is 18.11 °C, 8.39, and 1.23‰, respectively. Moreover, maximum growth rate (0.895–0.896) of *P. parvum* reached when the concentration of nitrogen, phosphorous, silicon and iron reach 3.41, 1.05, 0.69 and 0.53 mg/l, respectively. The order of the effects of the environmental factors impacting the biomass density of *P. parvum* was pH > salinity > water temperature, while the order of the effects of nutrients impacting the biomass density of *P. parvum* was nitrogen > phosphorous > iron > silicon. These findings may assist to implement control measures of the population of *P. parvum* where this harmful alga threatens aquaculture industry in the waterbodies such as Ningxia region in China.

*Prymnesium parvum* is a microscopic, planktonic, unicellular alga that belongs to the family Prymnesiaceae. Massive reproduction of *P. parvum* leads to harmful algal blooms globally, and causes accumulation of secreted cytotoxins, hemolytic toxins, and fish toxins in the waterbodies^1–4^. The accumulation of these toxins in the aquatic environment beyond a certain level will interfere with the normal physiological functions of the fish, and eventually the fish will die as they bleed out through deteriorated membranes of gill tissue. Although specific toxins of *P. parvum* have not yet been identified, they are thought to be certain type of fatty acid (e.g., linoleic acid) or a mixture of several fatty acids^5–7^. Since *P. parvum* can easily reproduce in waterbodies with high salinity, such as brackish water ponds and gulfs in the Northern China, production of these toxins has caused massive deaths of several farmed fish species. These large-scale fish kills have severe ecological and economic implications in this region.

Different algae have different nutrients requirements for their growth and development. This includes carbon (C), hydrogen (H), oxygen (O), nitrogen (N), phosphorus (P), potassium (K) and dozens of other macro and micro elements. However, nitrogen (N) and phosphorous (P) are the key macro nutrients that limit the growth of algae. The growth and morphology of algae is greatly affected by the different nitrogen (N) and phosphorus (P) mass concentrations and proportions^8^. Moreover, iron (Fe) is one of the most important micro elements involved in chlorophyll synthesis, electron transport, nitrogen fixation, photosynthesis, respiration and other metabolic processes of algae. The content and existing form of iron (Fe) not only affect the growth status of algae, but also affect the antioxidant capacity of algae^9,10^. Silicon (Si) is another essential micro nutrient for the growth of algae. In addition to its role as structural component of the cell wall, silicon is also involved in various metabolic and growth processes such as production of photosynthetic pigments and cell division^11^. In aquatic ecosystems, there is a complex interaction between these nutrients and their availability for algal growth^12^. The supply of certain nutrients to the algae will be restricted and affected by the presence of other nutrients^13^. Therefore, an optimum supply of nutrients is necessary to the growth of biomass density and population structure of this algae.

Ningxia region in China is characterized by saline and alkaline soils. Therefore, the water in the regional waterbodies, especially in Yinchuan and Yinbei, is highly alkaline (pH ranges from 7.8 to 9.2). Blooming of *P. parvum* has been intensively observed in the surface waterbodies in these regions. They flourish especially during the months when the water temperature is relatively low^14^. As a result, the toxins released by *P. parvum* often cause significant economic losses to the aquaculture industry and remarkably affect fishery production.

In this regard, we designed an experiment based on a uniform design to analyze the growth of *P. parvum* as affected by nutrients and environmental conditions. Uniform design is an experiment design method that combines number theories with multivariate statistics and puts forward on the basis of an orthogonal design, which has fewer experiments, low cost, and no loss of generality^15^. The advantages of a uniform design are reduction of number of experiments, short period of experimentation, high precision of the regression equation, and the ability to study the interaction between factors with minimal error^16^. The design requires that the number of tests arranged in a number of levels taken by the factors, so the number of levels taken by the factors can be increased appropriately, without worrying about the resulting increase in the number of tests squared like an orthogonal design.

Considering above, here we present a quasi-monte carlo method^17–19^ to quantify the effects of major nutrients (i.e., N, P, Si and Fe) and environmental factors (i.e., water temperature, pH and salinity) on the biomass density of *P. parvum*. We also constructed the growth model for *P. parvum* as affected by each of these nutrients and environmental factors to estimate optimum conditions of growth. We envisage that, understanding the growth patterns and optimum growing conditions of this environmentally harmful algae will guide to design mitigation measures through environmental modification and nutrient management in the affected waterbodies.

Aim: To quantify the effects of major nutrients and environmental factors on the biomass density of *P. parvum*.

## Methods

### Overview of the study area

The experimental algae *P. parvum* was collected from the fishponds in Dawukou, Ningxia, China. Algal water samples were filtered by medium size filter paper and centrifuged, and cultured with F/2 culture medium in the following environmental conditions for 5 days; light intensity of 5000 lx, light/dark ratio of 12 h: 12 h, water temperature of 18.5 ± 0.5 ℃, pH of 8.5 ± 0.1 and salinity of 1.2 ± 0.1 mg/l^20,21^. The plate separation method was used to separate and purify the cultured algae^22^. After microscopy, the colony of pure algal cells were transferred to different volumes of triangular glass bottles which contains sterilized F/2 medium for expansion culture. Algae *P. parvum* propagates vegetatively by cell division, the cell density of algae increases exponentially during the process of propagation thus requires more space. To accommodate this increasing space requirement, different sizes of the triangular glass bottles were used as 50 ml, 250 ml and 10 l. The expansion cultures were maintained in the environmental conditions similar to the initial culture. The algal cells were used for the experiment when they reach the logarithmic growth stage (the logarithmic growth stage was reached in 10 days).

### Data collection and experimentation

The water sample from the 10 L expansion culture of *P. parvum* was collected. The initial nutrient concentrations and environmental factors were determined using appropriate methods and equipment in the laboratory. The initial nutrient concentrations and environmental conditions of the algae culture used in this experiment is presented in Table 1.

**Table 1:** Initial nutrient concentrations and environmental conditions of algae culture used in the experiment.

Environmental condition/Nutrient concentration	Initial conditions
Water temperature (℃)	17.50
pH	8.50
Salinity (‰)	1.45
Nitrogen (mg/l)	3.68
Phosphorous (mg/l)	0.89
Silicon (mg/l)	0.81
Iron (mg/l)	0.62

Experimental factors and their levels for each nutrient concentrations and environmental factors were designed based on the above reference as shown in Table 2. We have designed eight levels for environmental factors (i.e., water temperature, pH and salinity) and ten levels for nutrient concentrations (i.e., nitrogen, phosphorous, silicon and iron).

1. Evaluation of the effects of environmental factors on the growth of *P. parvum*

**Table 2:** Experimental factors and their levels designed for the experiment.

Level	WT	pH	Salinity	N	P	Si	Fe
	(℃)		(‰)	(mg/l)	(mg/l)	(mg/l)	(mg/l)
1	9.0	7.0	0.8	2.0	0.2	0.2	0.1
2	11.0	7.3	1.0	2.3	0.4	0.4	0.2
3	13.0	7.6	1.2	2.6	0.6	0.6	0.3
4	15.0	7.9	1.4	2.9	0.8	0.8	0.4
5	17.0	8.2	1.6	3.2	1.0	1.0	0.5
6	19.0	8.5	1.8	3.5	1.2	1.2	0.6
7	21.0	8.8	2.0	3.8	1.4	1.4	0.7
8	23.0	9.1	2.2	4.1	1.6	1.6	0.8
9				4.4	1.8	1.8	0.9
10				4.7	2.0	2.0	1.0

*WT* water temperature; *N* nitrogen; *P* phosphorous; *Si* silicon; *Fe* iron.

To study the effects of environmental factors on the growth of *P. parvum*, water temperature, pH and salinity were used as the experimental factors by adopting the uniform design^23–25^ of three factors and eight levels as shown in Table 3.

**Table 3:** Combination of environmental factors used for the different levels in the uniform design.

Level	Environmental factors		
	WT (℃)	pH	Salinity (‰)
	X1	X2	X3
1	21.0	7.3	2.0
2	17.0	9.1	1.8
3	9.0	7.6	1.6
4	19.0	7.9	0.8
5	23.0	8.5	1.4
6	11.0	8.8	1.0
7	15.0	7.0	1.2
8	13.0	8.2	2.2

*WT* water temperature.

A 250 ml triangular glass bottle was used to implement each level of the above experiment with three replicates for each level (total of 24 bottles). The algae culture was allowed to grow in F/2 culture medium in the nutrient solution of 100 ml with an inoculation ratio of 1:10 (V/V). These bottles were kept in the light intensity of 5000 lx with light/dark ratio of 12 h: 12 h, while maintaining all other growth conditions to meet the experimental design requirements. The nutrient concentrations of N, P, Si and Fe were maintained at the level of initial concentrations (Table 1). Inoculated algae were cultured in a shaker for 10 days until it reaches its logarithmic growth stage and the growth rate was quantified.

2. Evaluation of the effects of nutrient concentrations on the growth of *P. parvum*

To study the effects of nutrient concentrations on the growth of *P. parvum*, nitrogen, phosphorus, silicon and iron were used as experimental factors by adopting the uniform design^5,26^ of four factors and ten levels as shown in Table 4. The culture medium was prepared with sodium nitrate (NaNO_3_) as the nitrogen source, monosodium phosphate (NaH_2_PO_4_) as the phosphorus source, sodium metasilicate (Na_2_SiO_3_) as the silicon source, and ferric citrate (FeC_6_H_5_O_7_) as a source iron to obtain the appropriate concentrations of nitrogen, phosphorous, silicon and iron as designed for this experiment (Table 2).

**Table 4:** Combination of nutrient concentration used for the different levels in the uniform design.

Level	Nutrient concentrations*			
	KNO_3_ (mg/l)	NaH_2_PO_4_ (mg/l)	Na_2_SiO_3_ (mg/l)	FeC_6_H_5_O_7_ (mg/l)
	X_i_	X_ii_	X_iii_	X_iv_
1	13.96(2.3)	2.32(0.6)	7.84(1.8)	0.88(0.2)
2	24.89(4.1)	1.55(0.4)	1.74(0.4)	3.94(0.9)
3	28.54(4.7)	3.10(0.8)	4.36(1.0)	1.31(0.3)
4	19.43(3.2)	0.77(0.2)	6.10(1.4)	2.19(0.5)
5	17.61(2.9)	6.97(1.8)	0.87(0.2)	1.75(0.4)
6	15.79(2.6)	6.19(1.6)	5.23(1.2)	4.38(1.0)
7	12.14(2.0)	3.87(1.0)	2.61(0.6)	3.06(0.7)
8	23.07(3.8)	5.42(1.4)	3.49(0.8)	0.44(0.1)
9	21.25(3.5)	4.65(1.2)	8.71(2.0)	3.50(0.8)
10	26.71(4.4)	7.74(2.0)	6.97(1.6)	2.63(0.6)

*Respective concentrations of nitrogen (N), phosphorous (P), silicon (Si) and iron (Fe) obtained from their primary sources are given in parenthesis.

A 250 ml triangular glass bottle was used to implement each level of the above experiment with three replicates for each level (total of 30 bottles). The algae culture was allowed to grow in F/2 culture medium with a volume of 100 ml and an inoculation ratio of 1:10 (V/V). These bottles were kept in the light intensity of 5000 lx, light/dark ratio of 12 h: 12 h, water temperature of 18.5 ± 0.5 ℃, pH of 8.5 ± 0.1 and salinity of 1.2 ± 0.1 mg/l. Inoculated algae were cultured in a shaker for 10 days until it reaches its logarithmic growth stage and the biomass density was quantified.

3. Determination of the growth rate of *P. parvum*

The algal cell density of the culture of each experimental level was measured using a 0.1 ml count plate under an optical microscope (Leica biological microscope DM1000, Leica Corporation, Oskar-Barnack-Straße, Germany) both at the beginning of the experiment and following 10 days of incubation period as the growth of the algae can reach its logarithmic growth stage at 10 days. Based on the algal cell density measurement, biomass density was calculated using the following formula (Eq. 1) described by Wei and Zhang;

1 $$ Growth\;rate\;\left( K \right) = 3.322 \times \left( {\log (N_{t} ) - \log \left( {N_{0} } \right)} \right)/\left( {t - t_{0} } \right) $$

where *t* is the duration of the experiment in days, *N*_0_ is the initial cell density (cell/ml) at the beginning of the experiment, and *Nt* is the cell density (cell/ml) at the end of day *t* of the experiment.

### Data analysis and results

1. Establishment of the regression model between environmental factors and the growth rate

**Figure 1:**
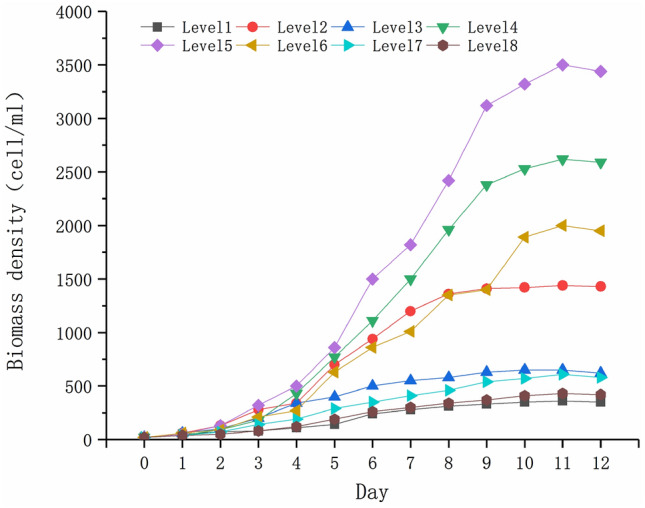
The growth curve of *P. parvum* with time under different environmental factor levels.

The growth rate of *P. parvum* under different levels of environmental factors are shown in Table 5, and the growth curve with time is shown in Fig. 1.

**Table 5:** Growth rates of *Prymnesium parvum* under the different levels of environmental factors in the uniform design.

Level	Environmental factors			
	WT (℃)	pH	Salinity (‰)	Growth rate
	X_1_	X_2_	X_3_	Y
1	21.0	7.3	2.0	0.410
2	17.0	9.1	1.8	0.615
3	9.0	7.6	1.6	0.502
4	19.0	7.9	0.8	0.698
5	23.0	8.5	1.4	0.738
6	11.0	8.8	1.0	0.656
7	15.0	7.0	1.2	0.480
8	13.0	8.2	2.2	0.438

*WT* water temperature.

In multiple quadratic stepwise regression analysis, water temperature (X_1_), pH (X_2_) and salinity (X_3_) were taken as independent variables, and the growth rate (Y) was taken as the dependent variable. From this analysis a quadratic polynomial regression equation (Eq. 2) was developed as follows:

2 $$ Y =  - 11.0371 + 0.0682X_{1}  + 2.5559X_{2}  + 0.7953X_{3}  - 0.0019X_{1} ^{2}  - 0.1523{\text{ }}X_{2} ^{2}  - 0.3223{\text{ }}X_{3} ^{2} $$

Correlation coefficient (R) of the above equation was 0.9994 and probability (*P*) of the regression equation was 0.025 (*p* < 0.05) as tested by the F-test, which indicates the significant relationship between the growth rate of *P. parvum* and the environmental factors. Therefore, the above regression model could robustly represent the relationship between the selected environmental factors and the growth rate of *P. parvum*. The standardized regression coefficients of each environmental factor of this model in the stepwise regression with the growth rate are shown in Table 6.

**Table 6:** The standardized regression coefficients of the growth model as affected by the environmental factors.

Environmental factor	WT (℃)	pH	Salinity (‰)
	X1	X2	X3
Standardized regression coefficient	2.7	15.0	3.1

*WT* water temperature.

Accordingly, the magnitude of the effect of each environmental factor on the growth rate was in the order of X_2_ > X_3_ > X_1_. Thus, the contribution of pH > salinity > water temperature on the growth rate of *P. parvum*.

2. Evaluation of the effect of environmental factors on the growth rate of *P. parvum*

The environmental conditions that would result in the maximum growth rate of *P. parvum* were determined by optimizing the regression equation (Eq. 2). The following simple regression models (Eqs. 3–5) of multiple quadratic stepwise regression analyses reveal the relationships between individual environmental factors and the growth rate. These models were obtained by dimensionality reduction analysis in which the other factors were maintained at optimal levels.

3 $$ X_{{1WT}} :\;Y\left( {X_{1} } \right) = 0.1768 + 0.0682X_{1}  - 0.0019X_{1} ^{2} $$

4 $$ X_{{2pH}} :\;Y\left( {X_{2} } \right) =  - 9.9345 + 2.5559X_{2}  - 0.1523{\text{ }}X_{2} ^{2} $$

5 $$ X_{{3salinity}} :\;Y\left( {X_{3} } \right) = 0.2982 + 0.7953X_{3}  - 0.3223{\text{ }}X_{3} ^{2} $$

**Figure 2:**
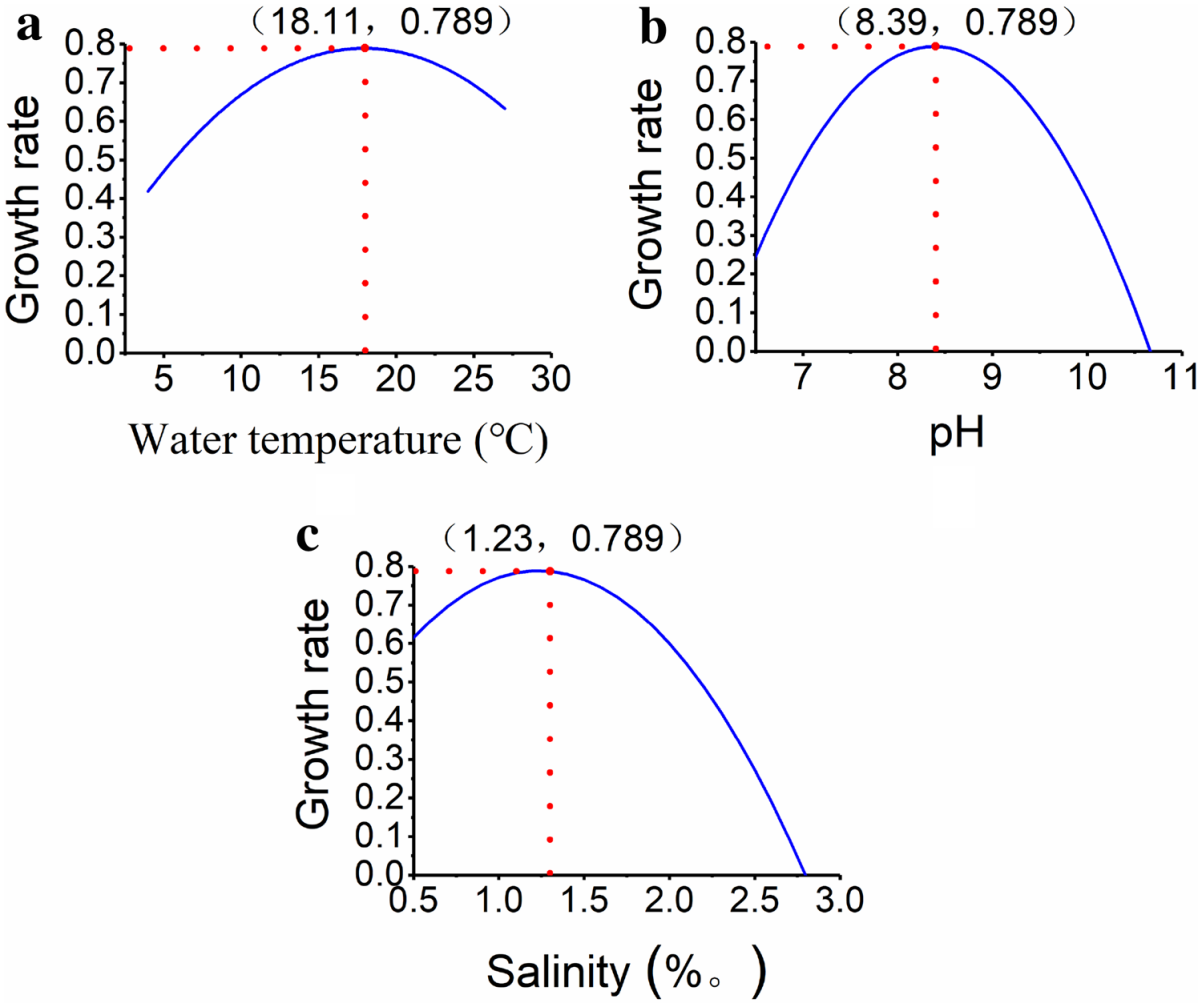
The growth rate of *P. parvum* as affected by the water temperature (**a**), pH (**b**) and salinity (**c**).

The influence curves of each environmental factor on growth rate of *P. parvum* are shown in Fig. 2. The behavior of the curves is similar where the growth rate increases initially, then reaches a theoretical maximum and finally declines with increasing level of each environmental factor. Accordingly, *P. parvum* reaches its theoretical maximum growth rate (0.789) when the water temperature, pH and salinity is 18.11 ℃, 8.39 and 1.23*‰*, respectively. Therefore, Fig. 2 can be considered as the growth model of *P. parvum* as affected each of the respective environmental factors.

3. Establishment of regression model between nutrient concentrations and the growth rate

**Figure 3:**
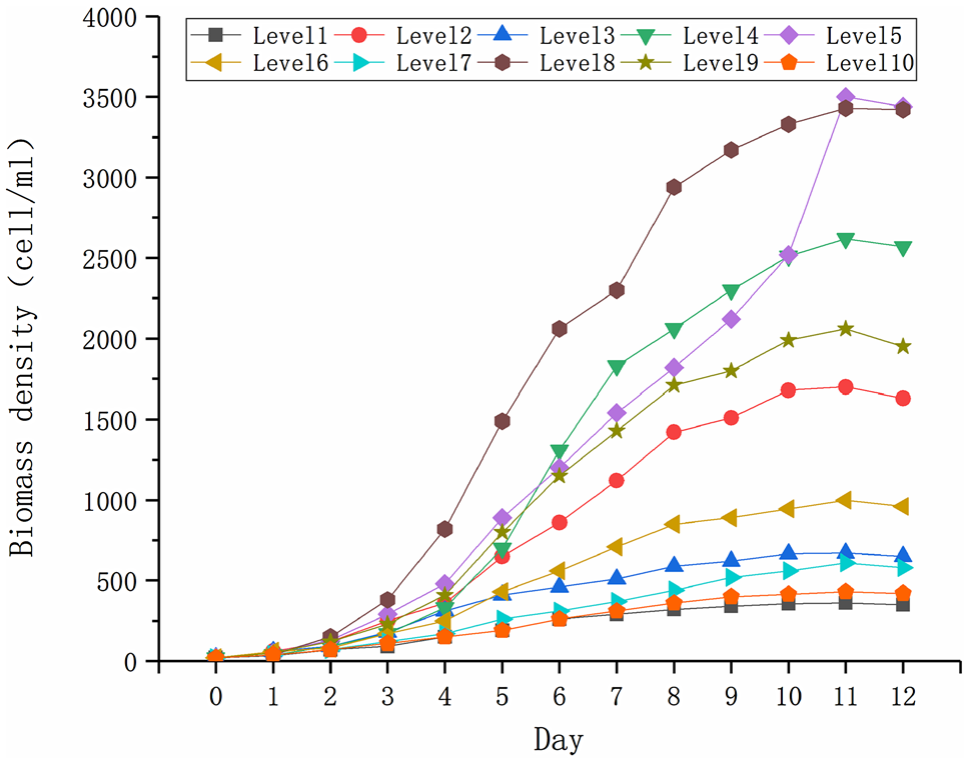
The growth curve of *P. parvum* with time under different nutrient concentrations factor levels.

The growth rates of *P. parvum* under the different levels of nutrient concentrations are shown in Table 7, and the growth curve with time is shown in Fig. 3.

**Table 7:** Growth rate of *Prymnesium parvum* under the different levels of nutrient concentration in the uniform design.

Level	Nutrient concentrations				
	KNO_3_(N)	NaH_2_PO_4_(P)	Na_2_SiO_3_(Si)	FeC_6_H_5_O_7_(Fe)	Growth rate
	X_i_	X_ii_	X_iii_	X_iv_	Y′
1	13.96(2.3)	2.32(0.6)	7.84(1.8)	0.88(0.2)	0.415
2	24.89(4.1)	1.55(0.4)	1.74(0.4)	3.94(0.9)	0.639
3	28.54(4.7)	3.10(0.8)	4.36(1.0)	1.31(0.3)	0.505
4	19.43(3.2)	0.77(0.2)	6.10(1.4)	2.19(0.5)	0.697
5	17.61(2.9)	6.97(1.8)	0.87(0.2)	1.75(0.4)	0.698
6	15.79(2.6)	6.19(1.6)	5.23(1.2)	4.38(1.0)	0.556
7	12.14(2.0)	3.87(1.0)	2.61(0.6)	3.06(0.7)	0.481
8	23.07(3.8)	5.42(1.4)	3.49(0.8)	0.44(0.1)	0.738
9	21.25(3.5)	4.65(1.2)	8.71(2.0)	3.5(0.8)	0.664
10	26.71(4.4)	7.74(2.0)	6.97(1.6)	2.63(.6)	0.437

A quadratic polynomial regression equation (Eq. 6) was generated using N (X_i_), P (X_ii_), Si (X_iii_) and Fe (X_iv_) as independent variables and the growth rate (Y′) as the dependent variable by using multiple quadratic stepwise regression analysis as follows:

6 $$ Y^{\prime }  =  - 1.856686 + 1.371680X_{i}  + 0.390361X_{{ii}}  + 0.150656X_{{iii}}  + 0.587990X_{{iv}}  - {\text{ }}0.2011178X_{i} ^{2}  - 0.186640{\text{ }}X_{{ii}} ^{2}  - 0.108764{\text{ }}X_{{iii}} ^{2}  - 0.550523{\text{ }}X_{{iv}} ^{2} $$

Correlation coefficient (R) of the above equation was 0.9994 and probability (*P*) of the regression equation was 0.035 (< 0.05) as tested by F-test, which indicates that the relationship between the growth rate of *P. parvum* and nutrient concentration is significant. Hence, the above regression model could robustly represent the relationship between the concentration of N, P, Si, Fe and the growth rate of *P. parvum*. The standardized regression coefficients of each nutrient in the main model (Eq. 6) of the stepwise regression with the growth rate are shown in Table 8.

**Table 8:** The standardized regression coefficients of the growth model as affected by the nutrient concentrations.

Nutrient concentration	N	P	Si	Fe
	X_i_	X_ii_	X_iii_	X_iv_
Standardized regression coefficients	10.5	2.0	0.8	1.5

Accordingly, the magnitude of the impact of each nutrient on the growth rate of *P. parvum* was in the order of X_i_ > X_ii_ > X_iv_ > X_iii_. Therefore, the contribution of nitrogen > phosphorous > iron > silicon for the growth of *P. parvum*.

4. Evaluation of the effect of nutrient concentrations on the growth rate of *P. parvum*

Multifactor square stepwise regression model was used to analyze the influence of individual nutrient concentration following the dimensionality reduction. To evaluate the influence of individual nutrient concentration on the growth rate, following sub-models (Eqs. 7–10) were developed by fixing other factors at the optimal level.

7 $$ X_{i} nitrogen:Y^{\prime } (X_{i} ) =  - 1.4432 + 1.3717X_{i}  - 0.2012X_{i} ^{2} $$

8 $$ X_{{ii}} phosphorus:Y^{\prime } (X_{{ii}} ) = 0.6916 + 0.3904X_{{ii}}  - 0.1866X_{{ii}} ^{2} $$

9 $$ X_{{iii}} silicon:Y^{\prime } (X_{{iii}} ) = 0.8436 + 0.1507X_{{iii}}  - 0.1088X_{{iii}} ^{2} $$

10 $$ X_{{iv}} iron:Y^{\prime } (X_{{iv}} ) = 0.7388 + 0.5880X_{{iv}}  - 0.5505X_{{iv}} ^{2} $$

**Figure 4:**
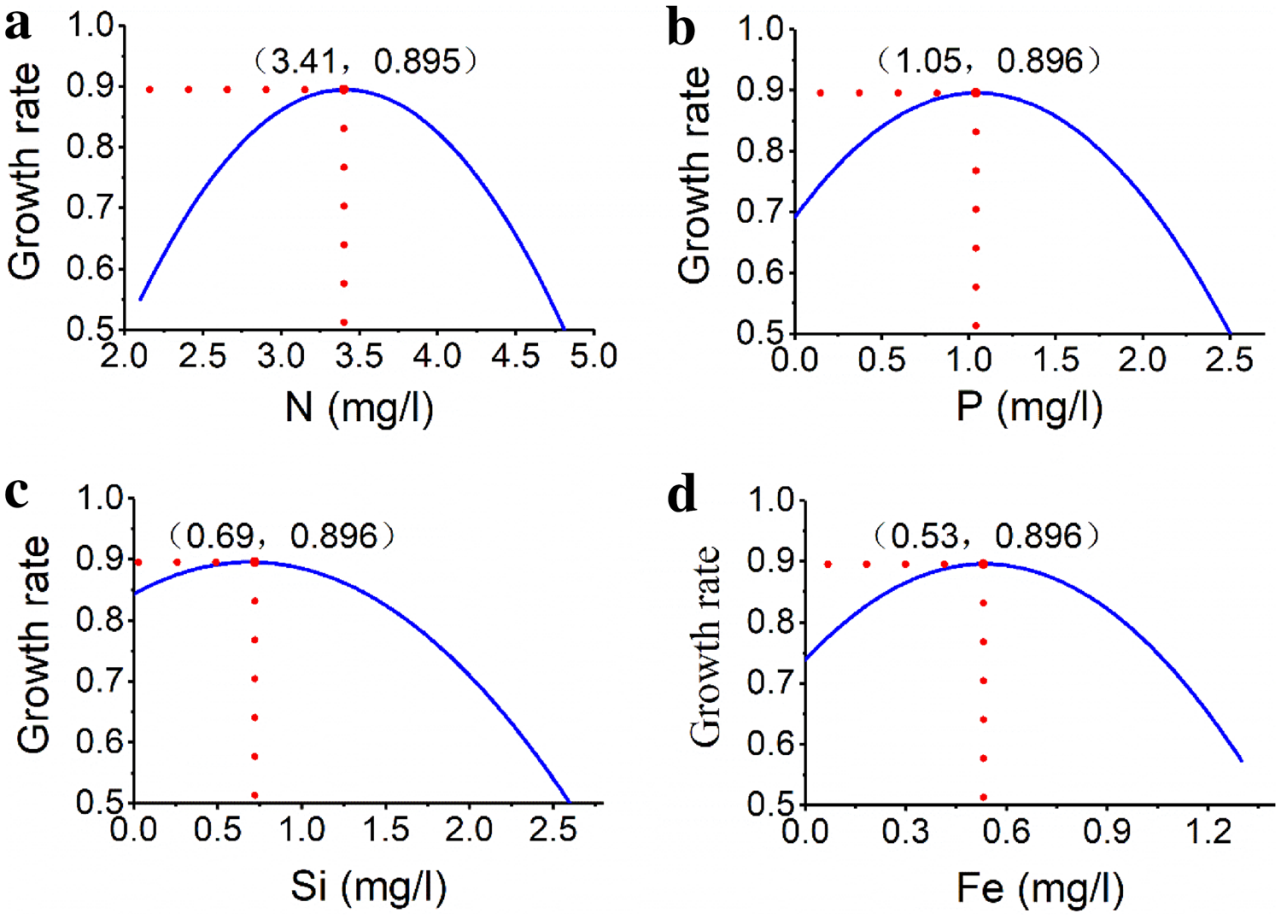
The growth rate of *P. parvum* as affected by nitrogen (**a**), phosphorus (**b**), silicon (**c**) and iron (**d**).

The influence curves of each nutrients on growth rate of *P. parvum* are shown in Fig. 4. The behavior of the curves shows an initial increase of the growth rate, then the growth rate reaches a theoretical maximum and finally declines with increasing level of concentrations of each nutrient. Accordingly, *P. parvum* reaches its theoretical maximum growth rate (0.896) when the concentration of nitrogen, phosphorous, silicon and iron is 3.41, 1.05, 0.69, 0.53 mgl^−1^, respectively. Therefore, Fig. 4 may be considered as the growth model of *P. parvum* as affected each of the respective nutrients.

## Discussion

1. Effect of water temperature

Temperature is one of the important environmental factors that affect the growth and reproduction of microalgae, and there are different degrees of influence on the activity of microalgae enzyme, the absorption and utilization efficiency of nutrients and the cell division^27^. In the certain temperature range, the higher the temperature, the greater the enzyme activity in the algal cells and the faster the metabolism rate. The more nutrients absorb, the quicker the algae grow. When the temperature exceeds the optimal growth range of the algae, the enzyme activity in the algae cells decreases or even loses its activity, which is not conducive to the respiration of the algae cells and the absorption of nutrients, thus affecting the growth of the *P. parvum*. The tolerance of microalgae to temperature varies between different species. Different algae have different temperature requirements and have their own suitable temperature range. The photosynthesis and respiration intensity of algae are affected by temperature, which affects the growth and development of algae and limits their distribution^28^. We found a significant regression relationship between the growth rate of *P. parvum* and water temperature. The growth rate of *P. parvum* increased as the water temperature increases but began to decrease when the temperature threshold was reached to 18.11℃ (Fig. 2a). This indicates that the water temperature beyond 18.11℃ could reduce the growth of *P. parvum*. In contrast, Baker^29^ found the occurrence of maximum growth at the temperature of 27 ℃, and the maximum cell concentration at 27 ℃, which were higher than the threshold water temperature values observed in our study. Moreover, optimal temperature for the growth of *P. parvum* often decreases with decreasing levels of salinity. This could be the reason for the lower threshold temperatures observed in this study.

2. Effect of pH

Water pH is an important ecological factor, which is closely related to the growth of algae^30^. Water pH mainly affects microalgae from two aspects. First, the growth of algae cells can be damaged by the changes in the acidity and alkalinity. Secondly, it affects the growth of algae by affecting the carbonate balance system and the distribution of different forms of inorganic carbon^31^. It also affects the absorption of nutrients and intracellular biochemical components for algae, which determines the activity of matrix and enzyme existing in different ionic forms^32,33^. The absorption of ammonium and nitrate is inhibited at high pH and semi saturated light intensity^34^. It is generally accepted that cyanobacteria prefer a higher pH^35^, and *Microcystis aeruginosa* has higher growth under pH range of 6.5–9.5. This study proved the significant regression relationship between the growth rate of *P. parvum* and pH. The growth rate of *P. parvum* increased as the pH increases but starts to decline when the pH exceeds 8.39 (Fig. 2b). This highlighted that excessively high pH (> 8.4) could reduce the growth of *P. parvum*.

3. Effect of salinity

Salinity is an important factor affecting the growth of any microalgae. Salinity affects the osmotic pressure, nutrient absorption, and suspension of algae. The increase or decrease of the salinity of the algae growth environment will lead to the increase or decline of osmotic pressure. The algal body regulates the osmotic pressure by regulating the ion concentration^36^, and the osmotic pressure regulation ability of different algae species is different. When the salinity conditions of the growth of the algae cells change, the osmotic pressure of cells change, thus cause damage to the algae cells. The change of salinity also affects the toxin synthesis ^37^. This study showed that, there was a significant regression relationship between growth rate of *P. parvum* and salinity. The growth rate of *P. parvum* increased as the salinity increases but began to decline when the salinity level reaches 1.23‰ (Fig. 2c). Therefore, our study shows that the salinity level greater than 1.23‰ could decrease the growth of *P. parvum*. Nonetheless, Baker^29^ presented that the maximum growth and maximum cell concentration of *P. parvum* occurs at 22 practical salinity units, which is substantially higher than the value observed in this study. However, the authors further argue that the bloom of *P. parvum* occurs at lower salinity and temperature levels, thus the acute toxicity to the fish would be higher at lower salinity and temperature levels. Therefore, the salinity and temperature levels used in our study would be more relevant to the problems caused by *P. parvum*.

4. Effect of nitrogen

Nitrogen is known as the key element of life and the main component of protein, nucleic acids, and phospholipids. Moreover, nitrogen is an essential nutrient for photosynthesis in algae cells. Lack of nitrogen can decrease chlorophyll synthesis in algae cells and impair the photosynthesis of algae cells. Additionally, high concentration of nitrogen may inhibit the growth of algae cells^38^. Different algae showed different preferences for the forms and the concentrations of nitrogen^38,39^. For example, Cyanobacteria prefers ammonia and the green algae prefers nitrate nitrogen^40^. Our study showed a significant regression relationship between the growth rate of *P. parvum* and the concentration of nitrogen^41^. The growth rate of *P. parvum* increased as the concentrations of N increases, but began to decrease as the respective concentration threshold was reached, which signifies that excessively high concentrations of N could affect the growth of *P. parvum* (Fig. 4a). We found that *P. parvum* reaches its maximum growth rate at the N concentration of 3.41mgl^−1^.

5. Effect of phosphorus

Phosphorus is a component of chloroplast bilayer membrane, grana, DNA and ATP of algae. It also plays an important role in material transformation of photosynthesis. It is one of the most important elements needed for microalgae growth and plays an important role in algal growth. The utilization rate of phosphorus is different for different microalgae^42^. The optimum phosphorus concentration of golden algae is 0.2 mg/L^43^, and *Nannochloropsis gaditana* is 1.1 mg/L^44^. This study showed a significant regression relationship between the growth rate of *P. parvum* and the concentration of P. The growth rate of *P. parvum* increased as the concentrations of P increases, but began to decrease as the respective concentration threshold was reached, which highlights that excessively high concentrations of P could reduce the growth of *P. parvum* (Fig. 4b). According to our growth model, *P. parvum* reaches its maximum growth rate at the P concentration of 1.05 mgl^−1^.

6. Effect of iron

Iron is the micronutrient and catalytic element of phytoplankton^45^. It is the component of oxidation–reduction carriers and coenzymes and involves in the process of chlorophyll biosynthesis and inorganic salt absorption in algal cells. Moreover, iron plays an important role in the process of cells oxidation and reduction^46^. Iron deficiency will affect many metabolic processes and even inhibit the growth of algal cell^47^. Our results showed a significant regression relationship between the growth rate of *P. parvum* and the concentration of Fe. The growth rate of *P. parvum* increased as the concentrations of Fe increases, but start to decline when the respective concentration threshold was reached, which indicates that excessively high concentrations of Fe could affect the growth of *P. parvum* (Fig. 4c). As per the growth model, *P. parvum* reaches its maximum growth rate at the Fe concentration of 0.53 mgl^−1^.

7. Effect of silicon

Silicon not only acts as a structural component of cell walls, but also participates in many metabolic and growth processes of algae such as synthesis of protein, photosynthetic pigments and DNA and cell division. Hence, silicon deficiency will affect the growth and development of algae^48^. This work showed a significant regression relationship between growth rate of *P. parvum* and the concentration of Si. The growth rate of *P. parvum* increased as the concentrations of Si increases, but began to decrease when the respective concentration threshold was reached, which highlights that excessively high concentrations of Si could interfere with the growth of *P. parvum* (Fig. 4d). The growth model indicates, *P. parvum* reaches its maximum growth rate at the Si concentration of 0.69 mgl^−1^.

The uniform design adopted in this experiment (Tables 3 and 4) is based on Quasi-Monte Carlo method, proposed by the Chinese mathematicians Fang Kai-Tai and Wang Yuan. This method can greatly reduce the number of tests, thus suitable for multi-factor and multi-level tests by making the test points uniformly dispersed on a high-dimensional space. Moreover, this method can create the limited data broadly representative^49^. Uniform design not only overcomes the inability of a single factor test to evaluate the effect of the interactions between factors, but also solve the problem of few factors in an orthogonal test. It has become a powerful tool for selecting optimal conditions in the search for the best experimental conditions and their interactions^50^. Therefore, under the condition of the same number of tests, more levels can be set by using uniform design, so that the test range of each factor can be further evaluated. Furthermore, uniform design has been effectively applied in the optimization of microalgae culture conditions. Hence, we adopted the uniform design to evaluate the effects of nutrients and environmental conditions on the growth of *P. parvum* and to model the growth of *P. parvum* as affected by selected nutrients and environmental factors.

Our study clearly showed the ability of applying uniform design to evaluate different environmental conditions and nutrients on the growth of *P. parvum*. Toxicity of *P. parvum* is directly affected by the physiological stress of algal cells when they undergo nutrient limitation^51^. Therefore, understanding the effects of essential nutrients the growth of *P. parvum* would be important to control the population dynamics of this environmentally harmful algae.

## Conclusions

In this study, we developed growth models of *P. parvum* and documented the optimal environmental conditions and nutrient concentrations for the growth of *P. parvum*. The uniform design was adopted to set up the experiment. Results have shown that the growth of *P. parvum* is significantly (*p* < 0.05) affected by environmental factors (water temperature, pH and salinity) and the availability of nutrients such as nitrogen, phosphorous, silicon and iron. The order of the magnitude of contribution of the environmental factors was pH > salinity > water temperature, while the order of the magnitude of contribution of the nutrient concentrations was nitrogen > phosphorous > iron > silicon on the growth of *P. parvum*.

Optimization analysis combined with the numerical simulation of one-way regression model, demonstrated that the *P. parvum* growth can reach the theoretical maximum of 0.789, when the water temperature, pH and salinity is 18.11 °C, 8.39, and 1.23‰, respectively. The same analysis for nutrient concentrations showed that the *P. parvum* growth rate could reach the theoretical maximum of 0.895–0.896, when the concentration of nitrogen, phosphorous, silicon and iron was 3.41, 1.05, 0.69 and 0.53 mg/l, respectively. This information is crucial to design and implement mitigation measures to control this environmentally harmful algae in the affected waterbodies.
